# Bewegung und Bewegungsförderung bei Menschen mit Migrationsgeschichte in Deutschland

**DOI:** 10.1007/s00103-026-04318-3

**Published:** 2026-10-19

**Authors:** Karim Abu-Omar, Antonina Tcymbal, Sven Messing, Peter Gelius

**Affiliations:** 1 Department für Sportwissenschaft und Sport, Friedrich-Alexander-Universität Erlangen-Nürnberg, Gebbertstr. 123b, 91058 Erlangen, Deutschland; 2 Physical Activity for Health Research Centre, Health Research Institute, Department of Physical Education and Sport Sciences, University of Limerick, Limerick, Irland; 3 Institut des Sciences du Sport, Université de Lausanne, Lausanne, Schweiz

**Keywords:** Körperliche Aktivität, Bewegung, Ungleichheit, Chancengerechtigkeit, Migration, Physical activity, Exercise, Inequality, Health equity, Migration

## Abstract

Gesundheitliche Chancengleichheit hat in der Bewegungs- und Gesundheitsförderung zunehmend an Bedeutung gewonnen und ist sowohl international als auch in Deutschland als wichtiges Ziel verankert. Da soziale Ungleichheiten und Mehrfachbenachteiligungen das Bewegungsverhalten beeinflussen, sollten Bevölkerungsgruppen mit besonderen Zugangsbarrieren, darunter Menschen mit Migrationsgeschichte, bei der Bewegungsförderung verstärkt berücksichtigt werden. Dieser Beitrag gibt einen Überblick über den Forschungsstand zu Bewegung und Bewegungsförderung bei Menschen mit Migrationsgeschichte in Deutschland.

Repräsentative Daten zeigen, dass Menschen mit Migrationsgeschichte seltener körperlich aktiv sind als Menschen ohne Migrationsgeschichte. Als zentrale Barrieren werden fehlende finanzielle Ressourcen, Sprachbarrieren, eingeschränkte Bewegungsgelegenheiten und kulturelle Faktoren berichtet. Dabei können sich verschiedene soziale Benachteiligungen, etwa aufgrund von Geschlecht, sozioökonomischer Lage und Migrationsgeschichte, überschneiden und Zugangsbarrieren verstärken. Am Beispiel des Projekts „Bewegung als Investition in Gesundheit“ (BIG) wird dargestellt, wie durch partizipativ entwickelte und zielgruppengerechte Angebote insbesondere Frauen mit Migrationsgeschichte erreicht werden können.

Für eine chancengerechte Bewegungsförderung sind neben partizipativen und zielgruppengerechten Angeboten auch strukturelle Rahmenbedingungen erforderlich, die den Zugang zu Bewegungsmöglichkeiten erleichtern. Die nachhaltige Implementierung und Skalierung entsprechender Maßnahmen können dazu beitragen, Zugangsbarrieren abzubauen und gesundheitliche Chancengleichheit zu fördern.

## Abstract (en)

Health equity has gained increasing importance in physical activity and health promotion and is firmly established as an important goal both internationally and in Germany. As social inequalities and multiple disadvantages influence physical activity behavior, population groups facing particular barriers to access—including people with a migration background—should receive greater consideration in physical activity promotion. This article provides an overview of the current state of research on physical activity and physical activity promotion among people with a migration background in Germany.

Representative data indicate that people with a migration background are less physically active than people without a migration background. Key barriers reported include limited financial resources, language barriers, restricted opportunities for physical activity, and cultural factors. Different forms of social disadvantage, for example those related to gender, socioeconomic status, and migration background, may intersect and reinforce barriers to access. Using the project “Bewegung als Investition in Gesundheit” (BIG; “Physical Activity as an Investment in Health”) as an example, this article illustrates how participatory and target-group-specific interventions can be used to reach, in particular, women with a migration background.

In addition to participatory and target-group-specific interventions, equitable physical activity promotion requires structural conditions that facilitate access to opportunities for physical activity. The sustainable implementation and scaling-up of such interventions can contribute to reducing barriers to access and promoting health equity.

## Einleitung

In der Bewegungs- und Gesundheitsförderung hat das Thema der gesundheitlichen Chancengleichheit in den letzten Jahren zunehmend an Bedeutung gewonnen. Dies wird beispielsweise im Globalen Aktionsplan für Bewegung der Weltgesundheitsorganisation (WHO) deutlich, in dem gesundheitliche Chancengerechtigkeit als Leitprinzip definiert wird [1]. Die WHO definiert gesundheitliche Chancengleichheit dabei als das Fehlen von ungerechten und vermeidbaren Unterschieden im Gesundheitszustand zwischen sozial, wirtschaftlich, demografisch oder geografisch definierten Bevölkerungsgruppen [2]. Auch in Deutschland wird im Nationalen Aktionsplan IN FORM das Ziel formuliert, vulnerable Bevölkerungsgruppen mit besonderem Unterstützungsbedarf verstärkt in den Blick zu nehmen [3]. Auch wissenschaftliche Studien zeigen, dass gesellschaftliche Ungleichheiten das Bewegungsverhalten beeinflussen. Beispielsweise zeigen frühere Analysen, dass sich in Deutschland Personen mit niedrigem Einkommen und niedriger Bildung sowie Personen mit Migrationshintergrund weniger bewegen [4].

Es scheint daher angezeigt, in der Bewegungsförderung einen besonderen Blick auf Menschen mit Migrationshintergrund zu legen. Zum einen machten sie laut Mikrozensus 2024 etwa 30 % der Gesamtbevölkerung in Deutschland aus [5]. Zum anderen weisen frühere Studien darauf hin, dass ein Migrationshintergrund ein relevantes, zugleich aber unscharfes Korrelat von Bewegung ist, also ein Merkmal, das mit Bewegungsverhalten zusammenhängt, ohne dieses zwangsläufig zu bestimmen. Relevant ist dieser Zusammenhang, weil eine Migrationsgeschichte mit vielen sozialen Benachteiligungen verbunden sein kann, die sich über unterschiedliche Wirkungswege auf das Gesundheitsverhalten und letztlich auf die Gesundheit auswirken können [6, 7].

Unscharf ist der Zusammenhang, weil eine Migrationsgeschichte nicht grundsätzlich mit gesellschaftlicher Benachteiligung einhergeht und Menschen mit Migrationsgeschichte eine sehr heterogene Bevölkerungsgruppe bilden. Die Zuordnung weist damit Merkmale eines sogenannten Fuzzy-Sets auf, also einer Gruppe, deren Grenzen und Zugehörigkeiten nicht immer eindeutig bestimmbar sind [8]. Auch vor diesem Hintergrund ist die Datenlage zu Bewegung, Bewegungsförderung und Migration in Deutschland uneinheitlich, gerade auch im Hinblick auf die berichteten Prävalenzen von körperlicher Aktivität.

Dennoch gibt es in der Praxis verschiedene Projekte und Initiativen, die sich explizit an Menschen mit Migrationsgeschichte richten. Die vom Bundesministerium für Gesundheit veröffentlichten Bestandsaufnahmen zu Bewegungsförderung in Deutschland nennen hier 3 konkrete Beispiele: (a) das Programm „Integration durch Sport“ des Deutschen Olympischen Sportbundes, das Menschen mit Migrationshintergrund dafür gewinnen will, sich aktiv auf allen Ebenen des Vereinslebens einzusetzen, (b) das Pilotprojekt „Gesundheitsförderung und Primärprävention bei älteren Menschen mit Migrationshintergrund“, in dem von 2007–2010 zielgruppenspezifische Präventionsmaßnahmen entwickelt wurden, und (c) das Projekt „Bewegung als Investition in Gesundheit (BIG)“, das sich seit 2005 an Frauen in schwierigen Lebenslagen richtet und dabei auch viele Frauen mit Migrationsgeschichte erreicht [9, 10].

Ziel dieses Beitrags ist es, einen Überblick über den derzeitigen Stand der Forschung zum Thema Migration und Bewegung in Deutschland zu geben. Dazu werden in einem ersten Schritt vorliegende Daten zum Bewegungsverhalten von Menschen mit Migrationsgeschichte in Deutschland vorgestellt. Danach werden die von ihnen berichteten Barrieren und Förderfaktoren in Bezug auf das Bewegungsverhalten zusammengefasst. Im dritten Schritt wird über das Projekt „Bewegung als Investition in Gesundheit“ (BIG) exemplarisch dargestellt, wie Bewegungsförderung bei Menschen mit Migrationsgeschichte in Deutschland gelingen kann.

Bei der Bearbeitung dieser Fragestellungen ist es ausdrücklich nicht die Intention des Beitrags „cultural othering“ [11] zu betreiben: Es geht nicht darum, herauszuarbeiten, dass oder wie Menschen mit Migrationsgeschichte bezüglich ihres Bewegungsverhaltens „anders“ sind, sondern vielmehr darum, darzustellen, welche besonderen Herausforderungen sie erfahren, um körperlich aktiv zu sein, und wie diesen Barrieren durch Maßnahmen auf gesellschaftlich-politischer Ebene wirksam begegnet werden kann.

### Begriffsbestimmungen.

Unter den verschiedenen Begriffen, die verwendet werden, um Menschen mit kulturellen Wurzeln in einem anderen Land zu beschreiben, ist der Ausdruck „Menschen mit Migrationsgeschichte“ der derzeit geläufigste und politisch empfohlene [12]. Der Begriff löst damit den vorher verwendeten des „Migrationshintergrunds“ zumindest teilweise ab. Wird Migrationshintergrund vom statistischen Bundesamt [13] verwendet, um Menschen zu charakterisieren, „wenn sie selbst oder mindestens ein Elternteil nicht mit deutscher Staatsangehörigkeit geboren wurde“, so wird diese Definition durch den Begriff der „Migrationsgeschichte“ auf Personen erweitert, „die rassistisch diskriminiert werden und denen ein Migrationshintergrund allgemein zugeschrieben wird. Diese Zuschreibung kann insbesondere an phänotypische Merkmale, Sprache, Namen, Herkunft, Nationalität und Religion anknüpfen“ [14]. Beide Begriffe gehen über die von den Vereinten Nationen verwendete Definition von Migration hinaus („Any person who is moving or has moved across an international border or within a State away from his/her habitual place of residence …“; [15]) und erkennen damit implizit an, dass Deutschland historisch ein Einwanderungsland ist.

Der Begriff der Migrationsgeschichte wird in diesem Beitrag verwendet, sofern es nicht konkret um Daten geht, die über das Konstrukt des Migrationshintergrunds gesammelt wurden. Für seine Nutzung spricht seine Konnotation, Migration als einen „normalen“ Teil der Gesellschaft anzusehen. Ebenso schließt er auch Menschen ein, die selbst keine Migrationserfahrung besitzen, denen diese aber zugeschrieben wird (z. B. durch alltägliche Diskriminierungen basierend auf der Hautfarbe). Und es ist zu konstatieren, dass auch durch diesen Begriff eine Verwendung zur Ausgrenzung und Stigmatisierung von Menschen nicht komplett vermieden werden kann.

## Beschreibung des methodischen Vorgehens

Für den Überblick zu Prävalenzen körperlicher Aktivität bei Menschen mit Migrationshintergrund in Deutschland wurden aktuelle repräsentative Studien aus Deutschland herangezogen. Berücksichtigt wurden Studien, die empirische Befunde zum Bewegungsverhalten, zur Sportbeteiligung oder zu Determinanten von körperlicher Inaktivität im Zusammenhang mit Migration berichten. Alle berücksichtigten Studien orientieren sich an dem Konstrukt des *Migrationshintergrundes* und nicht dem der *Migrationsgeschichte*. Die folgenden Studien und Datenquellen bilden die empirische Grundlage dieser Übersichtsarbeit:

- eine gepoolte Sekundäranalyse von Abu-Omar et al. von 13 nationalen Querschnittsstudien (1997–2018), in denen soziodemografische Determinanten von körperlicher Aktivität und Sportbeteiligung bei Erwachsenen ab 18 Jahren untersucht wurden [4];
- die GEDA-Fokus-Studie (Gesundheit in Deutschland aktuell: Fokus), die vom Robert Koch-Institut in den Jahren 2021/2022 durchgeführt wurde und querschnittlich das Bewegungsverhalten Erwachsener im Alter von 18 bis 79 Jahren mit kroatischer, italienischer, polnischer, syrischer oder türkischer Staatsangehörigkeit erfasste (*n* = 6038). Ziel dieser Studie war es, das Bewegungsverhalten sowie damit verbundenen soziodemografischen und migrationsbezogenen Faktoren zu erfassen [16];
- die KiGGS-Studie (Studie zur Gesundheit von Kindern und Jugendlichen in Deutschland), die Daten für Kinder und Jugendliche mit Migrationshintergrund enthält [17].

Die verwendeten Studien operationalisieren Migration zumeist anhand des Geburtslandes der Eltern (einseitiger vs. beidseitiger Migrationshintergrund), wobei sich die exakt verwendeten Definitionen zwischen den Datenquellen unterscheiden.

Die Literatur zu dem Thema Korrelate, Barrieren und Förderfaktoren des Bewegungsverhaltens bei Menschen mit Migrationsgeschichte wurde unsystematisch recherchiert und ausgewertet. Es wurde dabei sowohl auf einzelne nationale als auch internationale Studien zurückgegriffen, vornehmlich Reviews. Die Zusammenfassung der Studienergebnisse, die zumeist auf empirischen Erhebungen basieren, erfolgte narrativ. Darüber hinaus, um der Komplexität des Themas gerecht zu werden, sind auch einige eher theoretische Konzepte angeführt, die sich mit Problematiken von Anpassung und Fremdheit beschäftigen.

Das BIG-Projekt zur Bewegungsförderung bei Menschen mit Migrationsgeschichte wurde im Jahr 2005 vom Department für Sportwissenschaft und Sport der Friedrich-Alexander-Universität Erlangen-Nürnberg (FAU) initiiert. Ziel von BIG ist es, Bewegung bei sozial benachteiligten Frauen, insbesondere bei Frauen mit Migrationshintergrund zu fördern, die beispielsweise durch ein niedriges Haushaltseinkommen, Migrationsgeschichte, Arbeitslosigkeit, den Empfang von Sozialhilfe und/oder als alleinerziehende Mütter Barrieren zur Teilnahme an Bewegungsangeboten erfahren. Die Evaluation von BIG erfolgte in der Regel über den RE-AIM Ansatz (Reach, Effectiveness, Adoption, Implementation, Maintenance; [18]) und erlaubte damit eine Abschätzung der Reichweite, Wirksamkeit und Dauerhaftigkeit der Veränderungen auf der individuellen und strukturellen/verhältnisbezogenen Ebene.

Die dargestellten Daten zu der Teilnahmedauer an BIG-Bewegungsangeboten und den gesundheitlichen Wirkungen wurden über eine Querschnittsstudie erhoben. Da ein großer Teil der BIG-Teilnehmerinnen eine Migrationsgeschichte hatte und Deutsch häufig nicht die Muttersprache war, wurde die Befragung in 5 Sprachen (Deutsch, Englisch, Arabisch, Türkisch, Russisch) durchgeführt. Eine genauere Beschreibung des methodologischen Vorgehens findet sich unter Abu-Omar et al. [19].

## Prävalenzen des Bewegungsverhaltens

In allen untersuchten nationalen Datensätzen (1997–2018) berichteten Menschen mit Migrationshintergrund signifikant häufiger, fast nie oder nie Sport zu betreiben, als Menschen ohne Migrationshintergrund [19]. Die Odds Ratios (OR) für Nichtteilnahme an Sport lagen dabei je nach Datensatz und Einkommensquintil zwischen 1,81 und 3,70, was einer ungefähren Verdoppelung des Risikos entspricht, sportlich nicht aktiv zu sein.

### Erwachsene.

**Abb. 1:**
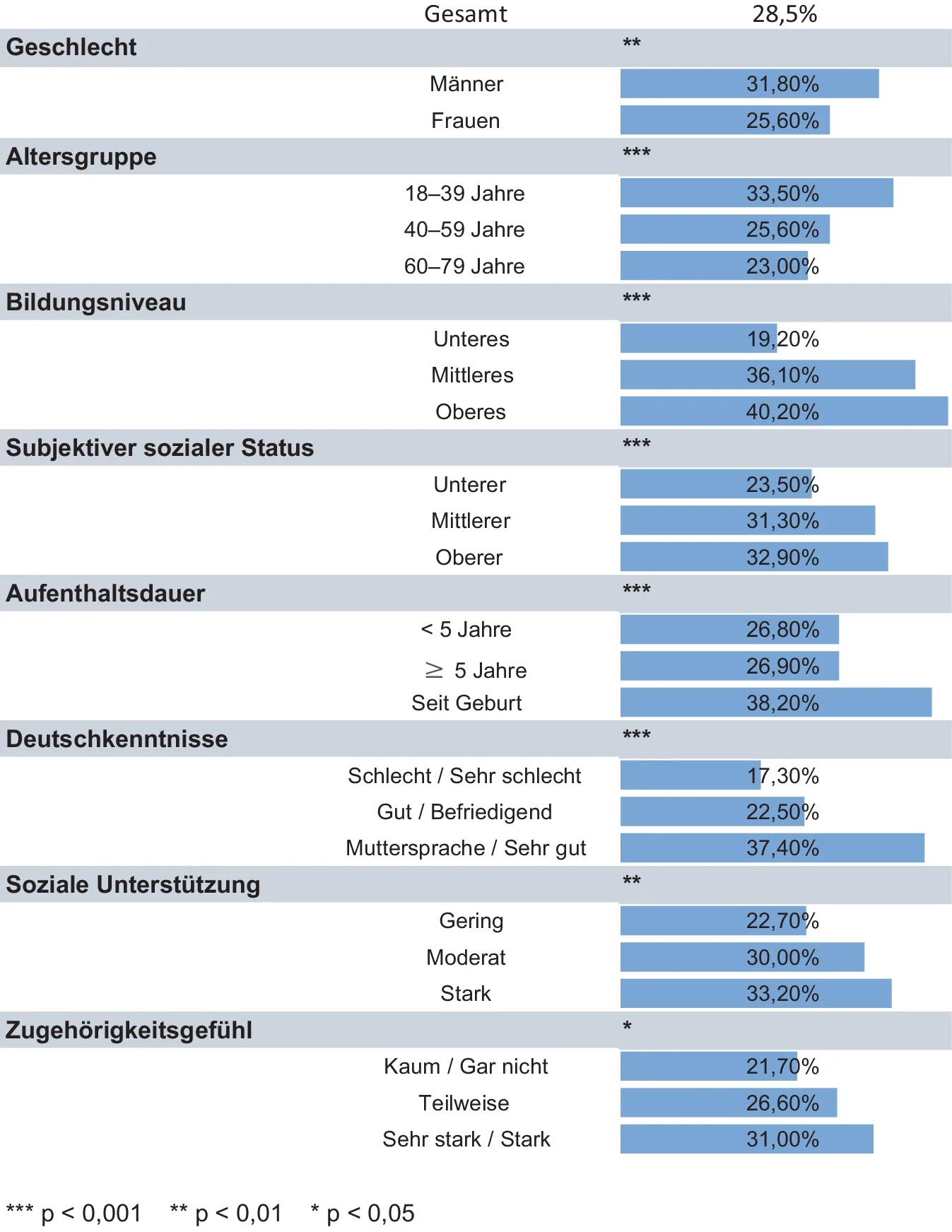
Prävalenz der Erreichung von Empfehlungen zur aeroben körperlichen Aktivität (≥ 150 min/Woche), Gesundheit in Deutschland aktuell(GEDA)-Fokus Studie 2021–2022. (Basierend auf den Ergebnissen von Zhang et al. [16])

**Abb. 2:**
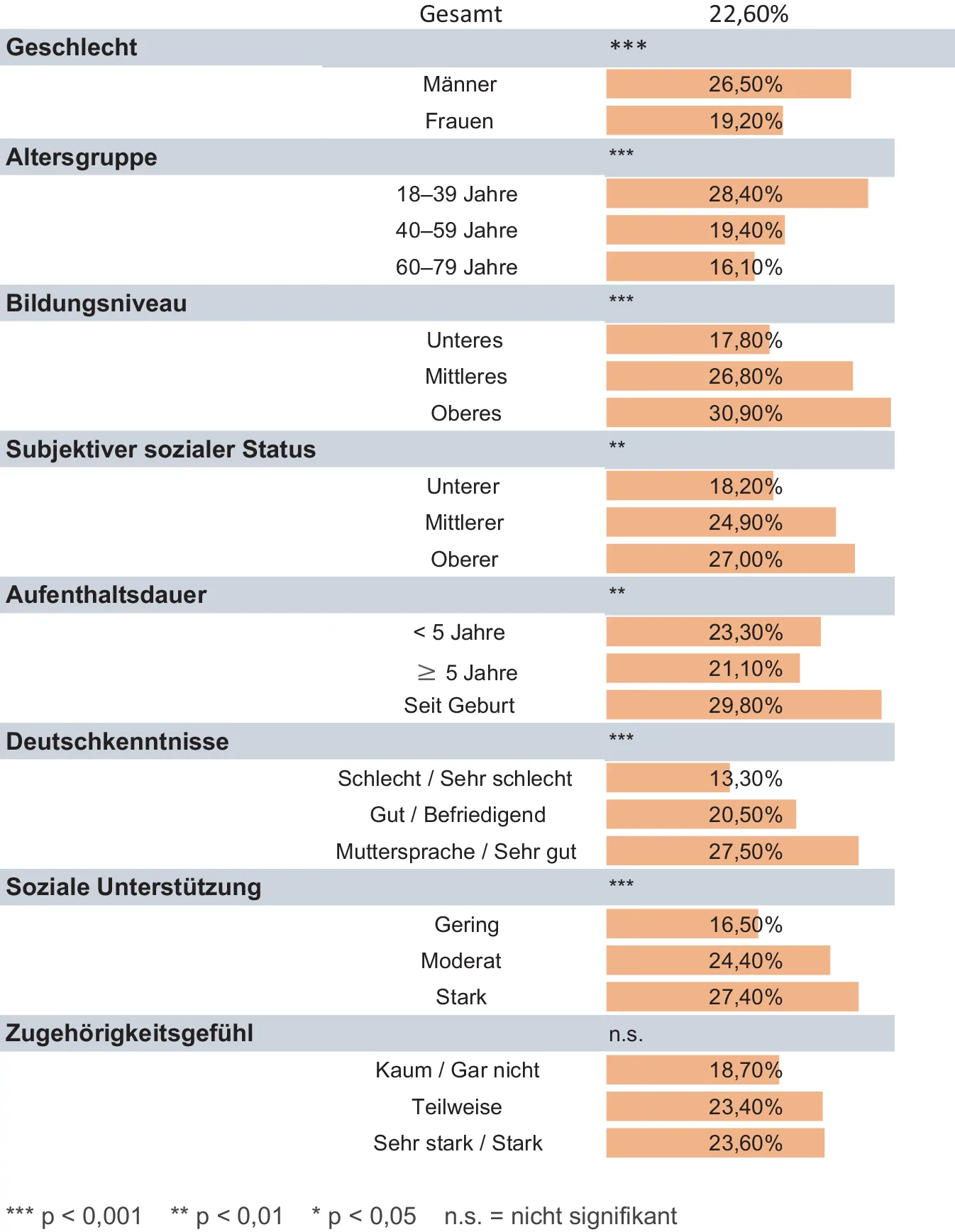
Prävalenz der Erreichung von Empfehlungen zur muskelkräftigenden Aktivität erreicht (≥ 2 Einheiten/Woche), GEDA-Fokus Studie 2021–2022. (Basierend auf den Ergebnissen von Zhang et al. [16])

Die GEDA-Fokus-Studie (2021–2022) liefert Schätzungen der Bewegungsprävalenzen speziell für Erwachsene mit Migrationshintergrund. Demnach erfüllen nur ca. 29 % unter ihnen die WHO-Empfehlungen für aerobe körperliche Aktivität (Abb. 1) und 23 % jene für muskelkräftigende Aktivitäten (Abb. 2). Multivariate Regressionsanalysen zeigen, dass Männer sowie Personen mit höherem Bildungsniveau, höherem subjektiven sozialen Status, besseren Deutschkenntnissen und höherem Ausmaß an sozialer Unterstützung häufiger die WHO-Empfehlungen für körperliche Aktivität erfüllen als die Vergleichsgruppen [15].

Auch in den Daten der GEDA-2019/2020-EHIS-Studien zeigt sich ein systematischer Unterschied in den Bewegungsprävalenzen zwischen Menschen mit und ohne Migrationshintergrund (Tab. 1). Diese Unterschiede liegen für unterschiedliche Bevölkerungsgruppen bei ca. 15–25 Prozentpunkten für die Merkmale Erfüllung der WHO-Empfehlungen zu aerober körperlicher Aktivität und Krafttraining.

**Tab. 1:** Prävalenz der Erreichung von Empfehlungen zur körperlichen Aktivität nach Geschlecht, Altersgruppe und Bildungsniveau

		Empfehlung zur aeroben körperlichen Aktivität erreicht (≥ 150 min/Woche), %		Empfehlung zur muskelkräftigenden Aktivität erreicht (≥ 2 ×/Woche), %	
		Allgemeinbevölkerung Deutschland (GEDA 2019/2020-EHIS)	Personen mit Migrationshintergrund (GEDA Fokus)	Allgemeinbevölkerung Deutschland (GEDA 2019/2020-EHIS)	Personen mit Migrationshintergrund (GEDA Fokus)
Total		48	28,5	36	22,6
Geschlecht	Männer	51	31,8	39	26,5
	Frauen	45	25,6	34	19,2
Altersgruppe	18–39 Jahre	57,7	33,5	45,7	28,4
	40–59 Jahre	46,8	25,6	33,5	19,4
	60–79 Jahre	38,2	23	30	16,1
Bildungsniveau	Unteres	35,1	19,2	29,8	17,8
	Mittleres	47,9	36,1	36,7	26,8
	Oberes	56,9	40,2	39,9	30,9

Über die Mitgliedschaft in Sportvereinen gibt es wenig aktuelle bevölkerungsrepräsentative Daten. Untersuchungen deuten aber an, dass hier eine differenzierte Betrachtung angemessen ist. So haben z. B. Einzelstudien zeigen können, dass in manchen Sportarten Männer mit Migrationsgeschichte in Sportvereinen überrepräsentiert sein können (z. B. Kampfsport, Fußball; [20]).

### Kinder und Jugendliche.

Bei Kindern und Jugendlichen zeigten die Daten der KiGGS-Studie (Welle 1, Motorik-Modul-Studie), dass Kinder und Jugendliche mit beidseitigem Migrationshintergrund im Vergleich zu Nichtmigranten sowie zu Kindern und Jugendlichen mit einseitigem Migrationshintergrund seltener in Sportvereinen Mitglied waren (56,2 % vs. 66,7 % bzw. 64,6 %) und seltener im Freien spielten (70,2 % vs. 78,7 % bzw. 75,7 %; [17]). Andere Studien zeigen in diesem Zusammenhang, dass Mädchen mit Migrationshintergrund deutlich seltener Mitglieder in einem Sportverein sind als Jungen [21]. Hingegen legten sie den Schulweg häufiger zu Fuß oder auf andere aktive Weise zurück (59,0 % vs. 47,8 % bzw. 52,9 %). Hinsichtlich der Teilnahme an außerschulischen körperlichen Aktivitäten sowie an körperlicher Aktivität außerhalb von Sportvereinen bestanden keine Unterschiede zwischen den 3 Gruppen.

Die Analyse der von Reimers et al. [17] mittels logistischer Regression zeigte, dass Kinder und Jugendliche mit beidseitigem Migrationshintergrund eine um 40 % geringere Chance haben, an Sportvereinsaktivitäten teilzunehmen (angepasstes OR = 0,60; 95 %-KI: 0,44–0,81). Bei Kindern mit einseitigem Migrationshintergrund war diese Chance 26 % geringer (angepasstes OR = 0,74; 95 %-KI: 0,55–1,00) im Vergleich zu Kindern ohne Migrationshintergrund. Bemerkenswerterweise hatten Kinder und Jugendliche mit beidseitigem Migrationshintergrund jedoch eine um 65 % höhere Wahrscheinlichkeit, an außerschulischer körperlicher Aktivität teilzunehmen, als Kinder ohne Migrationshintergrund (OR = 1,65; 95 %-KI: 1,15–2,36). Dies deutet darauf hin, dass Teilnahmeraten an Bewegung durchaus kontextspezifisch sind.

## Korrelate, Barrieren und Förderfaktoren des Bewegungsverhaltens

Ein vergleichsweise großer Korpus an Literatur berichtet über die Barrieren und Förderfaktoren, die Menschen mit Migrationsgeschichte im Bereich Bewegung erfahren. Bei den Barrieren kann dabei grundsätzlich zwischen strukturellen, sozialen und kulturellen und symbolischen Barrieren unterschieden werden [22]. Der Review von Caperchione et al. [23] bezieht sich auf Menschen mit Migrationsgeschichte aus kulturell und sprachlich diversen Ländern. Als mögliche Barrieren für Bewegung werden hier vor allem schwierigere sozioökonomische Lagen, schlechtere oder als nicht zugänglich empfundene Bewegungsgelegenheiten im Wohnumfeld, Sprachbarrieren sowie religiös und kulturell determinierte Einstellungen gegenüber dem persönlichen Gesundheitsverhalten bzw. dessen Einflussmöglichkeiten auf die eigene Gesundheit identifiziert.

Hinzu kommen auch Barrieren, die durch eine empfundene Sorge um die eigene Sicherheit entstehen, z. B. beim Aufenthalt in Naturräumen [18]. Dass diese Barrieren auch auf die Nachkommen von Menschen mit direkter Migrationsgeschichte wirken, erscheint nachvollziehbar. Paudel et al. (2023) haben die Barrieren untersucht, die Eltern mit Migrationsgeschichte für ihre Kinder berichten. Auch hier werden fehlende finanzielle Ressourcen und infrastrukturelle Möglichkeiten für Bewegung genannt, allerdings auch praktische Herausforderungen, wie z. B. Ermüdung der Eltern nach langen und anstrengenden Arbeitstagen [24]. Andere Studien, wie z. B. Lansburg et al. [25], bestätigen diese Befunde. Zorlu et al. [26] zeigen zudem auf, dass diese Barrieren auch in die dritte Generation hineinwirken, also die Enkelkinder der Zugewanderten.

Für Geflüchtete erweitert sich die Anzahl der Hinderungsfaktoren für Bewegung noch einmal deutlich. Gründe dafür sind einerseits die mit der Flucht verbundenen Erfahrungen und psychischen Belastungen [27], andererseits die insgesamt als prekär empfundene Lebenssituation in den Unterkünften für Geflüchtete [28]. Diese Barrieren gelten auch bei den Kindern von Geflüchteten [29] und sind auch für Deutschland belegt [30].

Ein Blick auf die Barrieren gibt umgekehrt auch Einsicht in mögliche Förderfaktoren für Bewegung bei Menschen mit Migrationsgeschichte: Als Förderfaktoren für Bewegung identifiziert wurden u. a. kultursensible Programme, die auf die Bedürfnisse von Menschen mit Migrationsgeschichte Rücksicht nehmen (z. B. nur für Frauen ausgelegt sind), kostenlos oder sehr preiswert sind, wohnortnah oder gut mit öffentlichen Verkehrsmitteln zu erreichen sind, Kleidung und Ausrüstung bereitstellen und unter Umständen auch Kinderbetreuung anbieten [31].

Über die Erkenntnisse dieser empirischen Arbeiten hinaus beschäftigen sich verschiedene sozialwissenschaftliche Ansätze mit der Komplexität von Anpassung, Fremdheit, Zugehörigkeit und Benachteiligung im Zusammenhang mit Migration. Dazu gehören z. B. die Konzepte der „körperlichen Fremdheit“ [32], des Belonging (Gefühl der Zugehörigkeit und sozialen Einbindung; [22]) und des Weathering (gesundheitliche Folgen einer langfristigen Belastung durch soziale Benachteiligung und Diskriminierung; [33]). Gemeinsam ist diesen Ansätzen, dass sie komplexe Wirkmechanismen beschreiben, die dazu führen können, dass Menschen sich von Angeboten des (Breiten‑)Sports nicht angesprochen oder bei deren Nutzung nicht willkommen fühlen.

## Bewegungsförderung bei Menschen mit Migrationsgeschichte – BIG als Beispielprojekt

Eine Vielzahl an Projekten zeigt Wege auf, wie es gelingen kann, Menschen mit Migrationshintergrund wirksam darin zu unterstützen, körperlich aktiv zu sein. Das Projekt „Bewegung als Investition in Gesundheit“ (BIG) ist nur eines von diesen. Bedingt durch die lange Laufzeit (das Projekt startete 2005 in Erlangen), den Transfer auf bisher 22 Kommunen und eine ausführliche wissenschaftliche Nachuntersuchung in den Jahren 2020–2023 mit einem Mixed-Method-Design dient [34] es an dieser Stelle als ein Beispiel guter Praxis. Ziel von BIG war es, körperliche Aktivität im Kontext von Sport und Freizeit bei Frauen in schwierigen Lebenssituationen (z. B. niedriges Einkommen, Migrationsgeschichte, Arbeitslosigkeit, Sozialhilfeabhängigkeit, Alleinerziehende) mithilfe eines partizipativen Ansatzes in der Tradition der „community-based participatory research“ zu fördern. Damit richtet es sich nicht ausdrücklich nur an Frauen mit Migrationsgeschichte. Als Herzstück dieses Ansatzes wurden Frauen in die Ausgestaltung niedrigschwelliger Bewegungsangebote (z. B. kostenlos, mit Kinderbetreuung, wohnortnah) aktiv eingebunden. Für die Beteiligung wurde dabei der Ansatz der „kooperativen Planung“ genutzt [35, 36], der Frauen, Forscher:innen und lokale Akteur:innen (z. B. Bürgermeister:innen, Sportvereinsvertreter:innen) gleichberechtigt einbezieht, Maßnahmen beschließt und anschließend ihre Umsetzung plant.

### BIG – Wirkungen auf der individuellen Ebene

Bewegungskurse, die im Rahmen des BIG-Projekts entwickelt wurden, zeichnen sich dadurch aus, dass sie durch den partizipativen Prozess genau auf die Bedürfnisse der Adressatinnen zugeschnitten sind. Dies bedeutet, dass Kurse kostenlos oder preisgünstig sind (zumeist 1–2 € pro Kurseinheit), häufig keine vorherige Registrierung benötigen, häufig kostenlose Kinderbetreuung anbieten, wohnortnah angeboten und kultursensibel angeleitet werden. Die von den Frauen gewünschten Bewegungsformen und Sportarten bilden ein weites Spektrum ab und sind mit den Angeboten zu vergleichen, die sich bei vielen Sportvereinen, Volkshochschulen oder Fitnessstudios finden.

Ein Merkmal von BIG ist die Vielfalt der über das Projekt erreichten Frauen über alle Standorte hinweg. Erhebungsdaten aus dem Jahr 2022 zeigten eine Altersspanne von 18 bis 84 Jahren, bei einem Durchschnitt von 48 Jahren. 32 % der Frauen gaben an, nicht die deutsche Staatsangehörigkeit zu besitzen, 29 % schätzten ihre Deutschkenntnisse als zufriedenstellend bis schlecht ein und 31 % gaben an, muslimischen Glaubens zu sein. Betrachtet man sozioökonomische Merkmale, so waren 42 % der Frauen berufstätig, 5 % arbeitssuchend und 9 % bezogen Sozialleistungen, wie z. B. Bürgergeld.

Die Vollerhebung unter den Teilnehmerinnen ermöglicht einen Einblick in die selbstberichteten Veränderungen des Bewegungsverhaltens der Frauen, die an BIG-Bewegungskursen teilnahmen. So gaben 54 % an, durch BIG mehr Sport zu treiben, 40 % beschrieben hingegen keine Veränderung ihres Bewegungsverhaltens. 34 % der Frauen gaben an, mindestens 2 h in der Woche Sport zu treiben. Die im Mittel berichtete Verweildauer in BIG-Kursen war mit 4 Jahren relativ hoch.

Neben Veränderungen des Bewegungsverhaltens berichteten die Frauen auch über Veränderungen anderer gesundheitsbezogener Verhaltensweisen. So gaben 32 % an, mehr auf ihre Ernährung zu achten. Nach den gesundheitlichen Wirkungen der Teilnahme an den BIG-Bewegungskursen gefragt, berichteten 53 % der Frauen von einer Verbesserung des Gesundheitszustands, 29 % sogar von einer erheblichen Verbesserung. Multivariate Analysen gepoolter BIG-Daten deuten zudem auf einen positiven Zusammenhang zwischen selbstberichteten guten Deutschkenntnissen und einer Verbesserung des Gesundheitszustands hin [37]. Positive Effekte von BIG auf die wahrgenommene soziale Unterstützung und die Entstehung neuer Freundschaften sind gerade auch durch die qualitativen Erhebungen gut belegt [38].

### BIG – Wirkungen auf der strukturellen Ebene

Der Transfer von BIG auf weitere Kommunen erfolgte mithilfe mehrerer aufeinanderfolgender Projektförderungen. Die durch die involvierten Wissenschaftler:innen eingeworbene Finanzierung (zumeist aus Mitteln von Landes- oder Bundesministerien oder den gesetzlichen Krankenkassen) zielte zum einen darauf ab, den Kommunen eine „Anschubfinanzierung“ für eine ca. 50 %-Personalstelle sowie weitere Mittel für den Aufbau von Bewegungskursen zur Verfügung zu stellen und zum anderen eine Begleitung und Evaluation von wissenschaftlicher Seite sicherzustellen.

Eine wichtige Einsicht aus dem Transfer von BIG ist, dass die wiederholt beobachteten Erfolge auf der individuellen Ebene nicht ohne Weiteres auf die strukturelle Ebene übertragbar sind. Vielmehr zeigt sich, dass nur in etwa 40 % der BIG-Standorte eine Verstetigung gelingt, wobei diese dann mit ca. 10 Jahren einen vergleichsweise langen Zeitraum umfasst. An anderen Standorten gelingt eine Verstetigung nur für deutlich kürzere Zeiträume von ca. 3–4 Jahren, bevor BIG aus verschiedenen Gründen von den beteiligten Organisationen aufgegeben wird. Qualitative Interviews mit den jeweiligen kommunalen Stakeholdern weisen auf unterschiedliche Ursachen hin: Veränderungen der politischen Schwerpunktsetzung in der kommunalen Gesundheitsförderung, finanzielle Schwierigkeiten der Kommunen oder aber der Wegfall der für die Organisation und Durchführung der BIG-Bewegungskurse verantwortlichen Person, beispielsweise infolge eines Arbeitsplatzwechsels oder des Eintritts in den Ruhestand [39]. Die große Bedeutung solcher „Champions“, d. h. besonders engagierter Personen, die die strukturelle Entwicklung und Umsetzung des Gesundheitsförderungsprojekts maßgeblich vorantreiben, ist auch aus anderen Studien zur Umsetzung von Maßnahmen der Gesundheitsförderung bekannt [40].

Ähnlich wie die Dauerhaftigkeit der BIG-Bewegungsangebote in einer Kommune ist auch deren Reichweite unterschiedlich. In einigen wenigen Kommunen wird BIG schon nach kurzer Zeit zu einem „vollen Erfolg“ mit jährlich steigenden Teilnehmerinnenzahlen. In anderen bleibt BIG trotz vieler Anstrengungen bei einer eher geringen Reichweite von wenigen Kursen [41] und scheint sich einem Wachstum zu widersetzen. Auch nach über 20 Jahren BIG-Transfererfahrung lässt sich aus wissenschaftlicher Sicht im Vorfeld nicht verlässlich prognostizieren, wie dauerhaft BIG in einer Kommune etabliert werden kann und welches Wachstumspotenzial dort besteht.

An dieser Stelle ist noch einmal zu betonen, dass viele weitere Projekte existieren, die Menschen mit Migrationshintergrund an Bewegungsangebote heranführen. So bietet zum Beispiel der Deutsche Olympische Sportbund (DOSB) über ca. 900 sogenannte Stützpunkvereine niederschwellige Sport- und Bewegungsangebote für Menschen mit Migrationsgeschichte und Geflüchtete an [28]. Ergebnisse einer Evaluation dieser Stützpunktvereine zeigen, dass es diesen gelingt, Menschen mit Migrationsgeschichte zu erreichen und zu integrieren [29]. Weitere Beispiele sind ein speziell an Geflüchtete gerichtetes Programm des Landessportbunds Berlin [42] sowie zahlreiche weitere vom Bundesamt für Migration und Flüchtlinge geförderte Projekte [43].

## Menschen mit Migrationsgeschichte – unscharfe Kategorie und unzureichende Datenbasis

Die vorgestellten quantitativen Daten zeigen, dass Migration in Deutschland mit einem geringeren Ausmaß körperlicher Aktivität assoziiert ist: Menschen mit Migrationsgeschichte scheinen sich seltener zu bewegen. In Studien, die das Konzept des Migrationshintergrunds verwenden, zeigen sich dabei Unterschiede zu Menschen ohne Migrationshintergrund von etwa 15–25 Prozentpunkten. Die genannten Barrieren für mehr Bewegung beziehen sich zum Teil auf individuelle Einstellungen, häufig aber auch auf als eingeschränkt wahrgenommene Bewegungsmöglichkeiten, fehlende finanzielle oder zeitliche Ressourcen sowie ein mangelndes Sicherheitsgefühlt. Projekten, die z. B. über Beteiligungsverfahren diese Barrieren abbauen und kultursensible Bewegungsangebote schaffen, scheint es zu gelingen, Menschen mit Migrationsgeschichte für Bewegung zu gewinnen.

Mit Blick auf diese Ergebnisse ist noch einmal zu betonen, dass die Analyse von Migration als Korrelat des Bewegungsverhaltens problematisch bleibt. So ist zu diskutieren, inwieweit es überhaupt sinnvoll ist, „Migrationsgeschichte“ als ein singuläres Korrelat des Bewegungsverhaltens herauszugreifen bzw. vereinfacht abzubilden. Entsprechende Analysen laufen immer Gefahr, leicht verfügbare, aber bei näherem Hinsehen wenig relevante Einzelmerkmale (z. B. die deutsche Staatsangehörigkeit) heranzuziehen und damit komplexe Zusammenhänge des Bewegungsverhaltens nur unzureichend abzubilden.

Trotz dieser durchaus berechtigten Kritik erscheint es ebenso wenig angemessen, Migration als Konstrukt aus Analysen der Gesundheits- und Bewegungsförderung auszuschließen. Dem stehen die vielfach berichteten Alltagserfahrungen von Menschen mit Migrationsgeschichte gegenüber, etwa im Zusammenhang mit ihrer Hautfarbe oder ihren Sprachkenntnissen, die mit erheblichen Zugangsbarrieren zu vielen bestehenden Bewegungs- und Sportangeboten in Deutschland verbunden sein können. Insofern bleibt Migrationsgeschichte wohl bis auf Weiteres eine unscharfe, aber dennoch relevante („fuzzy set“) Variable.

Zu dieser konzeptionellen Herausforderung kommt ein methodisches Problem hinzu: Menschen mit Migrationsgeschichte wurden in älteren Erhebungen zum Gesundheits- und Bewegungsverhalten in Deutschland häufig nur unzureichend berücksichtigt. Der Ausschluss erfolgte dabei zumeist nicht explizit, sondern implizit, indem für die Teilnahme an mündlichen oder schriftlichen Befragungen ausreichende Deutschkenntnisse eine Voraussetzung darstellten. Damit wurden Deutschkenntnisse zum entscheidenden Kriterium dafür, ob Menschen mit Migrationsgeschichte in den Daten überhaupt abgebildet wurden. Daher stellt sich die Frage, wie „repräsentativ“ diese Daten für Menschen mit Migrationsgeschichte in Deutschland überhaupt sind, gerade auch wenn man bedenkt, dass Deutschkenntnisse ein recht starker Prädiktor für das Bewegungsverhalten zu sein scheinen. Vor diesem Hintergrund überrascht es nicht, dass die Datenlage zu Gesundheitsverhalten und Gesundheitsstatus im Zusammenhang mit Migration noch 2015 vom Robert Koch-Institut als „rar“ beschrieben wurde [44].

Erst in jüngster Zeit, z. B. durch die Umfrage GEDA-Fokus und das Projekt „Improving Health Monitoring in Migrant Populations (IMIRA II)“, hat sich dieser Sachstand verbessert. Dennoch ist zu fragen, ob das Gesundheitsmonitoring in Deutschland in Zukunft nicht dahin gehend verändert werden sollte, dass eine Teilnahme in vielen verschiedenen Sprachen möglich wird. Darüber hinaus fällt auch auf, dass die großen bevölkerungsrepräsentativen Umfragen z. B. des RKI wenig Aussagen über die Mitgliedschaft in einem Sportverein bei Erwachsenen zulassen. Auch hier erscheint eine Verbesserung der Datenlage sinnvoll.

## Bewegungsförderung, Chancengleichheit und Intersektionalität

In der wissenschaftlichen Diskussion wird zunehmend die Auffassung vertreten, dass Bewegung als Menschenrecht verstanden werden sollte [45, 46]. Dies wird gerade auch mit Blick auf Geflüchtete diskutiert [47]. Aus einer solchen Perspektive werden Maßnahmen zur Schaffung bewegungsbezogener Chancengerechtigkeit noch dringlicher, gerade für Gruppen mit einem besonderen Schutzbedarf wie Geflüchtete [48].

Gesundheitsförderung und gesundheitliche Versorgung von Menschen mit Migrationshintergrund können darüber hinaus mit einem gesellschaftlichen und ökonomischen Nutzen verbunden sein, wie ein aktueller Bericht der Weltgesundheitsorganisation darlegt [49]. Für Interventionen der Bewegungsförderung wird neben gesundheitlichen und ökonomischen Effekten ein hoher sozialer Nutzen beschrieben [50]. So zeigte beispielsweise eine Studie von Loch et al. [51], dass ein verbesserter Zugang zu Grünflächen bei Menschen mit niedrigem Einkommen mit einer stärkeren Verringerung der Gesundheitskosten verbunden war als bei Menschen mit höherem Einkommen.

Für die Gesundheitsförderung haben in den letzten Jahren Theorien der Intersektionalität an Bedeutung gewonnen [52]. Intersektionalität beschreibt das Zusammenwirken verschiedener Faktoren, die mit Benachteiligung verbunden sein können (z. B. Geschlecht, sexuelle Orientierung, Hautfarbe), untereinander und mit strukturellen (Macht‑)Verhältnissen [53]. Gerade für das Bewegungsverhalten erscheint die Verwendung dieses theoretischen Bezugsrahmens sinnvoll, muss doch davon ausgegangen werden, dass solche multiplen Benachteiligungen die Barrieren für einen aktiven Lebensstil deutlich erhöhen [54]. So zeigen z. B. die Arbeiten von Mutz und Burrmann, dass Mädchen mit Migrationshintergrund deutlich seltener Mitglied in einem Sportverein sind als Jungen mit Migrationshintergrund [21]. Auch bei Bevölkerungsgruppen, bei denen sich verschiedene Formen der Benachteiligung überschneiden, kann die partizipative Entwicklung von Maßnahmen dazu beitragen, multiple Zugangsbarrieren zu Bewegungsgelegenheiten systematisch abzubauen.

Neben dem Konzept der Intersektionalität können aber auch Konzepte wie das der „körperlichen Fremdheit“ erklärend wirken. Gerade für den Sport ist gut herausgearbeitet worden, wie Bewegungsmuster, die in anderen Kulturkreisen erlernt und z. B. in Sportvereinen praktiziert werden, Gefühle der Fremdheit hervorrufen können, weil sie von der Mehrheit als „ungewöhnlich“ wahrgenommen werden [32].

## Fazit

Die Auseinandersetzung mit Migration wirft zugleich Fragen nach den zukünftigen Strukturen der Gesundheits- und Bewegungsförderung in einer „postmigrantischen Gesellschaft“ [36] auf, also einer Gesellschaft, in der Fragen der Zugehörigkeit, Partizipation und eben auch Chancengerechtigkeit von Menschen mit Migrationsgeschichte ausgehandelt werden. Damit verbunden ist dann auch die große Herausforderung, wie die Gesundheits- und Bewegungsförderung mit migrationsfeindlichen gesellschaftlichen Diskursen umgehen kann.

Die vorliegenden Erkenntnisse zeigen, dass Bewegungsförderung bei Menschen mit Migrationsgeschichte gut gelingen kann. Dazu gehören beispielsweise Maßnahmen zur Schaffung von Infrastrukturen, die den Zugang zu Bewegungsmöglichkeiten verbessern, sowie öffentliche Investitionen in aktive Mobilität, etwa in Radwege oder Fußgängerzonen in sozial benachteiligten Stadtteilen. Ebenso zu nennen sind sogenannte Whole-Systems-Ansätze, bei denen Bewegungsförderung nicht durch einzelne Maßnahmen, sondern durch das koordinierte Zusammenwirken verschiedener Akteure, Strukturen und Maßnahmen in einer Lebenswelt umgesetzt wird. In Schulen können solche Ansätze neben der Bewegung auch das Gesundheitsbewusstsein von Kindern und Jugendlichen stärken [55]. Eine zentrale Voraussetzung für Chancengerechtigkeit besteht darin, die finanziellen Mittel und strukturellen Rahmenbedingungen für eine nachhaltige Implementierung und Skalierung solcher Maßnahmen bereitzustellen.

## Data Availability

Für diesen Artikel wurden keine Datensätze erstellt oder analysiert.
